# Solid type primary intraosseous squamous cell carcinoma in a cat

**DOI:** 10.1186/s12917-018-1344-0

**Published:** 2018-01-22

**Authors:** Darja Pavlin, Tamara Dolenšek, Tanja Švara, Ana Nemec

**Affiliations:** 1 University of Ljubljana, Veterinary faculty, Small Animal Clinic, Gerbičeva, 60 Ljubljana, Slovenia; 2 University of Ljubljana, Veterinary faculty, Institute of Pathology, Wild Animals, Fish and Bees, Gerbičeva, 60 Ljubljana, Slovenia

**Keywords:** Primary intraosseous squamous cell carcinoma, Squamous cell carcinoma, Cat, Odontogenic oral tumors

## Abstract

**Background:**

Squamous cell carcinoma (SCC) is the most common nonodontogenic oral tumor in cats. In the jaw, it usually presents as an ulceroproliferative lesion associated with enlargement of the affected bone.

**Case presentation:**

This report describes the case of a cat in which clinical and radiographic findings of a mandibular swelling were suggestive of an aggressive process, but the oral mucosa was unaffected. The results of histopathological and immunohistochemical examination of the samples obtained from the intraosseous lesion were consistent with SCC. The animal was euthanized 5 months after initial presentation as a result of the severe progression of the disease, and no other primary tumors were identified at necropsy.

**Conclusions:**

Based on the clinicopathological, microscopic, and immunohistochemical staining features, as well as the absence of a primary tumor at a distant site, we propose that the term, solid type primary intraosseous SCC (PIOSCC), be used to describe this neoplasia, as it shares similar features with human PIOSCC.

## Background

Mandibular swelling can occur in cats as a result of tumors, osteomyelitis or cysts [1, 2]. Of the oral tumors, squamous cell carcinoma (SCC) is the most common tumor, representing approximately 60–70% of all feline oral malignancies [3]. SCC is a tumor of older cats with a mean age at presentation of 12.5 years [4]. There is no known breed or sex predisposition in cats.

SCC is a nonodontogenic oral tumor of epithelial origin, typically presenting as an ulceroproliferative lesion in the sublingual area or on the mandibular or maxillary gingiva, which is associated with enlargement of the affected bone. Other less common sites include the buccal mucosa, lips and pharynx. Characteristic clinical signs include excessive salivation, hemorrhagic or purulent oral discharge, pain, difficulty eating and tooth loss. Radiographs frequently reveal significant bone lysis, although new bone formation can also be observed. Due to the rapid and invasive growth of these tumors, SCC in cats is often very advanced at the time of initial presentation [4]. The previously presumed low metastatic rate of these tumors appears to be an underestimate, since up to 37.5% of these tumors metastasized to regional lymph nodes in a study by Gendler et al. [5]. However, the majority of cats die due to the primary disease prior to development of clinically evident metastatic disease [3]. Despite considerable efforts to develop effective treatments, prognosis for feline patients with oral SCC is poor, since none of the therapeutic options currently available are curative or result in long-term control of the disease [4]. The median survival time (MST) with palliative treatment (nonsteroidal anti-inflammatory drugs, antibiotics, and corticosteroids) is approximately 1.5 months [3, 4]. Surgery, when feasible, increases the MST to 6 months, which can be extended to up to approximately one year with multimodal therapy (different combinations of surgery, radiotherapy and chemotherapy) [4].

In humans with swelling of the jaw, primary intraosseous squamous cell carcinoma (PIOSCC) is considered as a possible differential diagnosis. PIOSCC is a very rare invasive tumor, and in contrast to the more common “classical” oral SCC, it is an odontogenic tumor. It develops within the mandible or maxilla without any initial connection to the oral mucosa. The diagnostic criteria, differentiating PIOSCC from other similar tumors (e.g., oral SCC, alveolar carcinoma, or metastatic bone lesions), include undamaged oral mucosa and the absence of a primary tumor at a distant site. The WHO classification categorizes PIOSCC into three subtypes: solid de novo tumors that originate from remnants of odontogenic epithelium or, rarely, dedifferentiation from a benign ameloblastoma, tumors originating from odontogenic cysts and those originating from keratocystic odontogenic tumors [6].

## Case presentation

A 14-year-old 3.5 kg female spayed strictly indoor domestic shorthair cat was admitted to the Small animal clinic, Veterinary faculty Ljubljana, Slovenia, for evaluation of a facial skin lesion of approximately two weeks duration. The history and general physical examination were unremarkable, except for a small superficial autotraumatic skin lesion in the right mandibular region (ventrally at the level of the right mandibular canine tooth). A brief dermatologic examination of the cat revealed no other abnormalities of the skin or coat and no evidence of ectoparasites. The right rostral mandible appeared swollen. On a brief oral examination, several missing teeth, a severely mobile right mandibular canine tooth and moderate generalized plaque, calculus and gingivitis affecting the remaining of the teeth were noted.

**Fig. 1:**
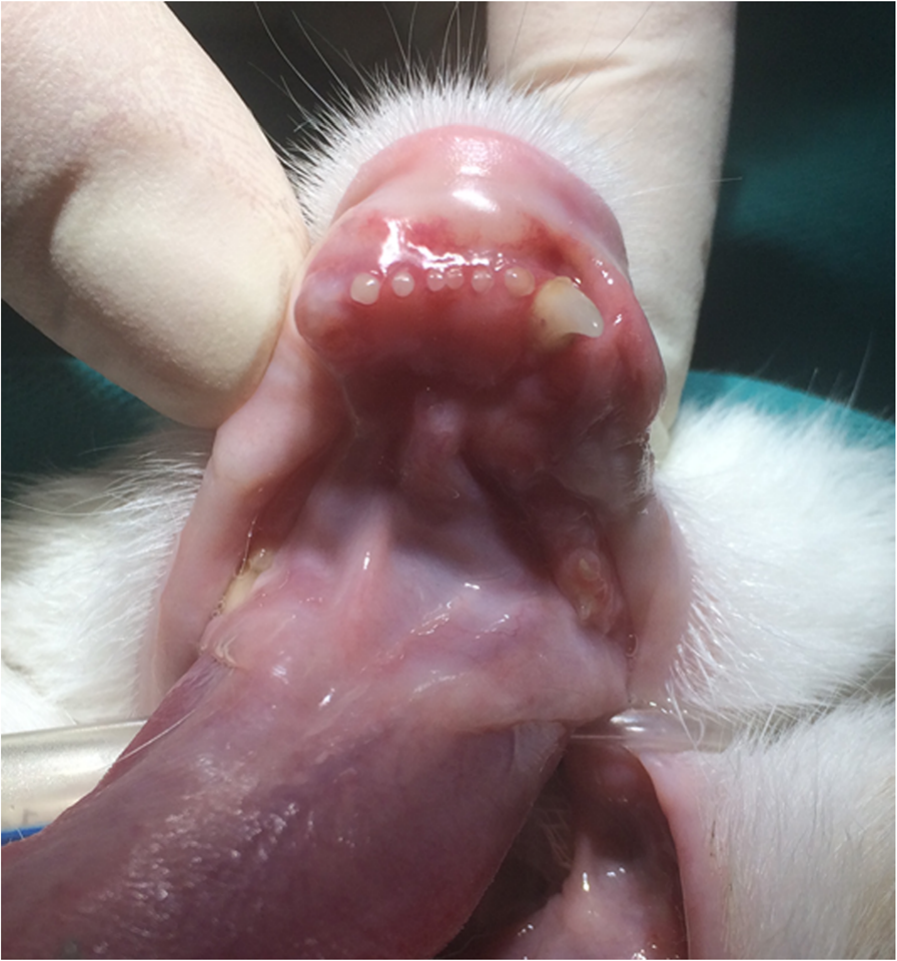
The rostral mandibles of the cat in dorsal recumbency under general anesthesia. Swelling of the rostral mandibles is notable, but there is no oral soft tissue lesion

**Fig. 2:**
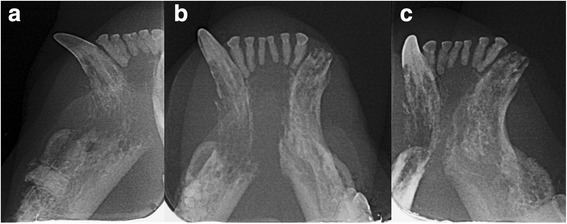
Right lateral (**a**), occlusal (**b**) and left lateral (**c**) dental radiographs of the rostral mandibles of the cat. Geographic bone loss is evident at the right rostral mandible and symphyseal area, combined with permeative bone loss in the apical region of the right mandibular canine tooth. Mild inflammatory root resorption is present in all of the incisor teeth. The left mandibular canine and third premolar tooth are affected by stage 5 tooth resorption. The right mandibular canine tooth is affected by stage 4c tooth resorption and there is a complete loss of hard tissues in the apical area. The right mandibular third premolar tooth is affected by stage 5 tooth resorption and there is a retained mesial root of the right mandibular fourth premolar tooth

Informed consent was obtained from the client to perform a detailed oral and dental examination under general anesthesia with dental radiographs and biopsy, as indicated. A basic preanesthetic bloodwork panel was within normal limits (Table 1). The three-view thoracic radiographs were unremarkable and other diagnostic imaging procedures (e.g., CT scan) were declined by the owner. The detailed oral and dental examination revealed swelling and palpable instability of the rostral mandibles with severe mobility of all of the mandibular incisor teeth and the right mandibular canine tooth. No excessive probing depth, gingival recession or any other soft tissue lesions were diagnosed for any of the teeth (Fig. 1). Several teeth were missing, gingivitis was present on all of the remaining teeth and tooth resorption of various stages was diagnosed at several of the remaining teeth. The dental radiographs of the rostral mandibles are presented in Fig. 2. The mandibular lymph nodes were palpably within normal limits.

**Table 1:** Preanesthetic bloodwork results

Parameter	Result	Reference value
Complete blood count		
WBC	5.02 × 10^9^/L	6.3–19.6
% Neutrophils	66.2	29.5–74.5
% Lymphocytes	27.7	20.0–61.2
% Eosinophils	2.3	3.4–11.4
% Monocytes	3.8	0.2–5
% Basophils	0	0–1.0
RBC	8.95 × 10^12^/L	6.0–10.1
Ht	0.45	0.28–0.47
PLT	219 × 10^9^/L	156.4–626.4
Pct	0.31	0.3–0.8
Biochemistry		
Urea	8.9 mmol/L	5.3–12.1
Creatinine	126 μmol/L	70.7–140

*WBC* white blood cells, *RBC* red blood cells, *Ht* hematocrit, *PLT* platelets, *Pct* plateletcrit

**Fig. 3:**
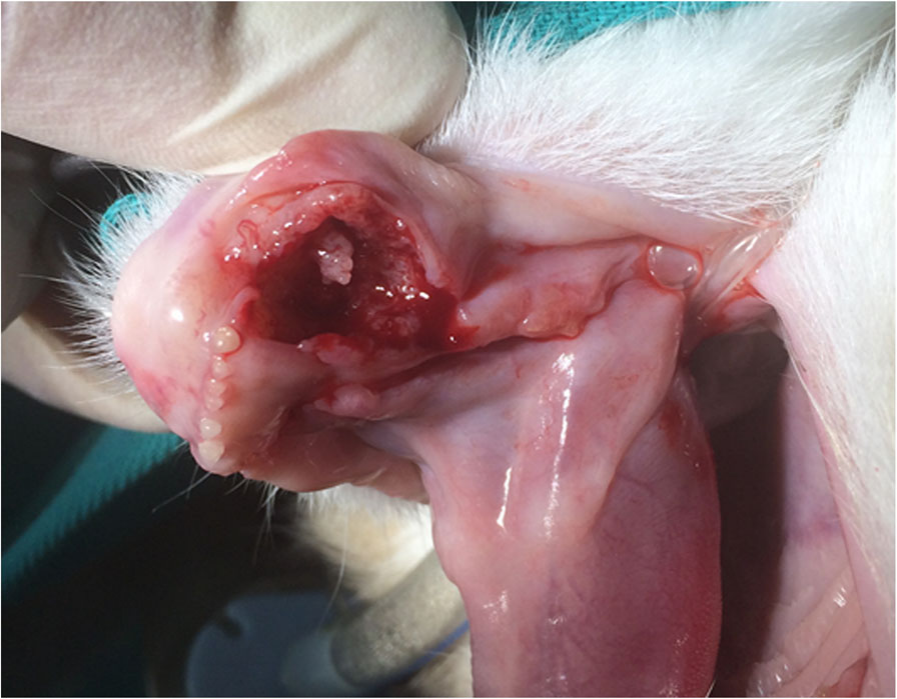
An intraoperative photograph of the right rostral mandible after a full-thickness triangular flap was created and the crown of the right mandibular canine tooth was removed. Proliferative soft tissue is visible filling the alveolus of the right mandibular canine tooth

Based on the clinical and radiographic features, an aggressive process, such as osteomyelitis or cancer, was suspected, and a biopsy was recommended. The oral cavity was rinsed with a 0.12% chlorhexidine solution, and left and right inferior alveolar nerve blocks were performed with 0.2 ml of 2.5 mg/ml levobupivacaine prior to performing a professional dental cleaning. A full-thickness triangular flap was created to remove the remnants of the right mandibular canine tooth and to obtain soft tissue and bony samples for histopathology (Fig. 3). The flap was sutured back in place with 5–0 resorbable monofilament suture material, and other dental treatments were postponed pending the biopsy results. The cat was discharged from the hospital with oral meloxicam (0.1 mg/kg/day), which was to be administered once daily until the re-check examination.

The incisional biopsies that were collected for histopathology were fixed in 10% buffered formalin, embedded in paraffin, sliced into 4 μm sections and stained with hematoxylin and eosin stain. Samples containing a large amount of bony tissue were decalcified with OSTEOMOLL^®^ (Merck Millipore) before further processing.

**Fig. 4:**
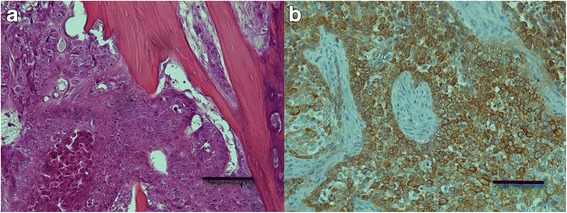
Primary intraosseous squamous cell carcinoma (PIOSCC) of the mandible in the cat. **a** Sheets of polygonal neoplastic cells with eosinophilic, nongranulated cytoplasm and round to oval nuclei with prominent nucleoli are evident infiltrating the bony tissue. A focus of dyskeratotic neoplastic cells is present in the left bottom corner. HE. Bar = 100 μm **b** The neoplastic cells exhibit a moderate to marked positive cytoplasmic reaction for cytokeratin. Immunohistochemistry for cytokeratin. Bar = 100 μm

The histopathological examination of the biopsies revealed an infiltrative lesion, composed of islands and cords of oval to polyhedral cells, which exhibited marked anisocytosis and had lightly eosinophilic, nongranulated cytoplasm and round to oval nuclei with moderate anisokaryosis and one to two prominent nucleoli. The mitotic index was 20 mitotic figures per 10 high power fields. Some of the neoplastic cells were binucleated or trinucleated. Foci of dyskeratotic neoplastic cells were evident, but true keratinization was not present. There was a moderate amount of fibrous stroma, which was multifocally infiltrated with lymphocytes. The neoplastic cells infiltratively grew into the surrounding bony tissue, but no blood or lymph vessel invasion was noted (Fig. 4a).

Additionally, immunohistochemistry was conducted on formalin-fixed, paraffin-embedded tissue sections to confirm the epithelial origin of the neoplastic cells. Mouse monoclonal antibody raised against human cytokeratin (clone MNF116; Dako, Glostrup, Denmark), which was diluted 1:100, was used for the immunolabelling. The antigen retrieval was performed by microwave treatment at medium power (550 W) for 20 min in a 0.1 M citrate buffer (pH 6.0). The remaining immunohistochemical procedure was performed using a previously described protocol [7]. Sections of normal feline skin were used as a positive control, and sections not treated with primary antibodies served as a negative control. Immunohistochemically, a moderate to marked positive cytoplasmic reaction for cytokeratin was observed in almost all of the neoplastic cells (Fig. 4b).

**Fig. 5:**
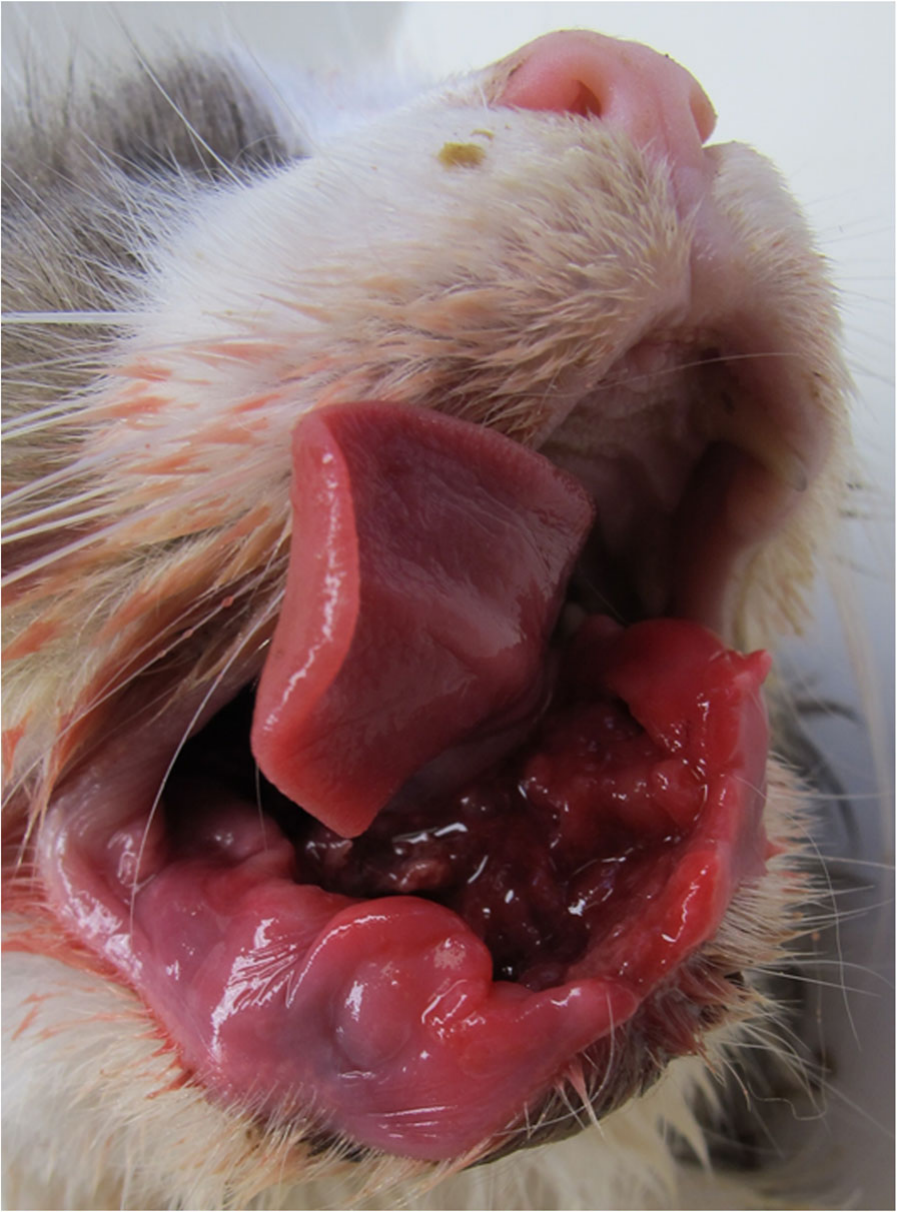
The rostral mandibles of the cat at necropsy. The majority of the right rostral mandible, symphysis and a 1.5 cm long segment of the left rostral mandible are severely thickened with the right rostral mandible demonstrating segmental osteolysis. The lower lip is severely swollen and deformed. There is extensive and deep mucosal ulceration extending into the sublingual mucosa

A two-week re-check examination revealed progression of clinical signs with a more pronounced mandibular swelling. Soft tissue proliferation at the biopsy site and hemorrhagic oral discharge were present at this time. The client declined further procedures and elected palliative pain medications. Five months after the initial presentation, the client elected humane euthanasia of the animal due to the rapid deterioration of its health. An extensive oral lesion was found on necropsy (Fig. 5) and the mandibular lymph nodes were mildly to moderately enlarged. No other tumors were detected elsewhere in the body. Histopathology revealed multiple islands of carcinomatous cells bilaterally in the mandibular lymph nodes, but no other primary tumor or metastases were discovered.

## Discussion and conclusions

Based on the clinicopathological, microscopic, and immunohistochemical staining features and the absence of a primary tumor at a distant site, we propose that the lesion in this cat be diagnosed as a solid type PIOSCC. In human medicine, PIOSCC is a rare oral tumor, representing less than 2% of all oral SCCs in people [8–10]. The majority of PIOSCCs arise from other benign odontogenic tumors or cysts, while solid de novo type PIOSCC is extremely rare [11, 12]. Although PIOSCC has not yet been described in cats, in the clinical case presented here, the radiographic and microscopic features were very similar to those described for human solid type PIOSCC. Namely, the cat presented with very non-specific clinical signs in which the major complaint was an autotraumatic superficial skin lesion on the chin. Given the findings of the extensive oral examination, it was considered likely that the lesion was a result of pain or discomfort arising from the oral cavity. Although solid type PIOSCCs in humans are frequently asymptomatic, pain and jaw swelling without oral soft tissue involvement, as observed in this cat, is considered to be the main clinical features of solid type PIOSCC in humans [6, 13, 14].

The proposed diagnosis of solid type PIOSCC in this cat was further supported by the radiographic findings, which revealed an osteolytic lesion associated with tooth resorption; this outcome was similar to the typical radiographic appearance of human solid type PIOSCC [6, 15, 16].

The histopathological findings were consistent with oral SCC in cats [17] and SCC [18] and solid type PIOSCC in humans [6]. Given the clinicopathological features in this cat, and since prominent features including cellular atypia, moderate mitotic activity and no true keratinization were observed in the sections examined; this tumor was further classified as a poorly differentiated nonkeratinizing solid type PIOSCC [13, 19, 20].

Immunohistochemically, a moderate to marked positive cytoplasmic reaction for pancytokeratin was observed in almost all of the neoplastic cells. Cytokeratins are intermediate filaments found in epithelial cells of all types and are therefore specific markers for an epithelial cell lineage [21]. This immunohistochemical finding confirmed an epithelial origin of the tumor in our case.

Although there have already been five cases reported in the literature describing SCC in cats that presented with mandibular swelling without oral soft tissue lesions [22, 23], there is an important difference in the radiographic appearance of our case and the previously described cases. The skull radiographs performed in three of the previously described five cases exhibited a predominant mixed pattern of osteoproduction and osteolysis mimicking osteosarcoma. Quigley et al. even described a “sunburst appearance” of the affected mandible, while our case demonstrated only an osteolytic lesion radiographically [22]. Additionally, the microscopic appearance of our case differs from those previously described cases. Both of the previous reports emphasized osteoblastic and fibrous proliferation, whereas in our case, these features were absent. This leads us to believe that our case differs from the previously reported cases in cats in some important respects and may therefore represent a different pathological entity. Since the pathological entity described in our case resembles the features of human solid type PIOSCC, we propose that the term solid type PIOSCC be used to describe this condition in cats.

## Abbreviations

MST: median survival time
PIOSCC: Primary intraosseous squamous cell carcinoma
SCC: Squamous cell carcinoma

## References

[1] Kapatkin AS, Manfra Maretta S, Patnaik AK, Burk RL, Matus RE (1991). Mandibular swellings in cats: prospective study of 24 cats. J Am Anim Hosp Assoc.

[2] LaDouceur EE, Walker KS, Mohr FC, Murphy B (2014). Odontogenic keratocyst in a cat. J Comp Pathol.

[3] Bilgic O, Duda L, Sánchez MD, Lewis JR (2015). Feline oral squamous cell carcinoma: Clinical manifestations and literature review. J Vet Dent.

[4] Mc E, FJM V, Lommer MJ (2012). Clinical behavior of nonodontogenic tumors. Oral and maxillofacial surgery in dogs and cats.

[5] Gendler A, Lewis JR, Reetz JA, Schwartz T (2010). Computed tomographic features of oral squamous cell carcinoma in cats: 18 cases (2002 – 2008). J Am Vet Med Assoc.

[6] Eversole LR, Siar CH, van der Waal I, Barnes L, Evson JW, Reichart P, Sidransky D (2005). Primary intraosseous squamous cell carcinomas. World Health Organization Classification of Tumors. Pathology and Genetics Head and Neck Tumors.

[7] Cociancich V, Gombač M, Švara T, Pogačnik M (2013). Malignant Mesenchymoma of the aortic valve in a dog. Slov Vet Res.

[8] Jing W, Xuan M, Lin Y, Wu L, Liu L, Zheng X (2007). Odontogenic tumours: a retrospective study of 1642 cases in a Chinese population. Int J Oral Maxillofac Surg.

[9] Adebayo ET, Ajike SO, Adekeye EO (2005). A review of 318 odontogenic tumors in. Kaduna, Nigeria. J Oral Maxillofac Surg.

[10] Naruse T, Yanamoto S, Sakamoto Y, Ikeda T, Yamada SI, Umeda M (2016). Clinicopathological study of primary intraosseous squamous cell carcinoma of the jaw and a review of the literature. J Oral Maxillofac Surg.

[11] Bodner L, Manor E, Shear M, van der Waal I (2011). Primary intraosseous squamous cell carcinoma arising in an odontogenic cyst: a clinicopathologic analysis of 116 reported cases. J Oral Pathol Med.

[12] Saxena C, Aggarwal P, Wadhwan V, Bansal V (2015). Primary intraosseous squamous cell carcinoma in odontogenic keratocyst: a rare entity. J Oral Maxillofac Pathol.

[13] Chaisuparat R, Coletti D, Kolokythas A, Ord RA, Nikitakis NG (2006). Primary intraosseous odontogenic carcinoma arising in an odontogenic cyst or de novo: a clinicopathologic study of six new cases. Oral Surg Oral Med Oral Pathol Oral Radiol Endod.

[14] Choi YJ, Oh SH, Kang JH, Choi HY, Kim GT, Yu JJ (2012). Primary intraosseous squamous cell carcinoma mimicking periapical disease: a case report. Imaging Sci Dent.

[15] Alotaibi O, Al-Zaher N, Alotaibi F, Khoja H, Qannam A (2016). Solid-type primary intraosseous squamous-cell carcinoma in the mandible: report of a rare case. Hematol Oncol Stem Cell Ther.

[16] Matsuzaki H, Katase N, Matsumura T, Hara M, Yanagi Y, Nagatsuka H (2012). Solid-type primary intraosseous squamous cell carcinoma of the mandible: a case report with histopathological and imaging features. Oral Surg Oral Med Oral Pathol Oral Radiol.

[17] Head KW, Cullen JM, Dubielzig RR (2003). Histological classification of tumors of the alimentary system of domestic animals.

[18] Johnson N, Franceschi S, Ferlay J, Ramadas K, Schmid S, MacDonald DG, Barnes L, Evson JW, Reichart P, Sidransky D (2005). Squamous cell carcinoma. World Health Organization classification of tumors. Pathology and genetics of head and neck tumors.

[19] Huang JW, Luo HY, Li Q, Li TJ (2009). Primary intraosseous squamous cell carcinoma of the jaws. Clinicopathologic presentation and prognostic factors. Arch Pathol Lab Med.

[20] Wenguang X, Hao S, Xiaofeng Q, Zhiyong W, Yufeng W, Qingang H (2016). Prognostic factors of primary intraosseous squamous cell carcinoma (PIOSCC): a retrospective review. PLoS One.

[21] Painter JT, Clayton NP, Herbert RA (2010). Useful immunohistochemical markers of tumor differentiation. Toxicol Pathol.

[22] Quigley PJ, Leedale A, Dawson IM (1972). Carcinoma of mandible of cat and dog simulating osteosarcoma. J Comp Pathol.

[23] Takagi S, Mori T, Watanabe K, Kadosawa Tm Ochiai K, Trigoe S (2004). Mandibular squamous cell carcinoma with reactive bone proliferation in two cats. Jpn J Vet Anesth & Surg.

